# Insect-Induced Daidzein, Formononetin and Their Conjugates in Soybean Leaves

**DOI:** 10.3390/metabo4030532

**Published:** 2014-07-04

**Authors:** Shinichiro Murakami, Ryu Nakata, Takako Aboshi, Naoko Yoshinaga, Masayoshi Teraishi, Yutaka Okumoto, Atsushi Ishihara, Hironobu Morisaka, Alisa Huffaker, Eric A Schmelz, Naoki Mori

**Affiliations:** 1 Graduate School of Agriculture, Kyoto University, Kitashirakawa, Sakyo, Kyoto 606-8502, Japan; E-Mails: murakami.shinichiro.85e@st.kyoto-u.ac.jp (S.M.); nakata.ryu.ski@gmail.com (R.N.); frecher@kais.kyoto-u.ac.jp (T.A.); yoshinaga.naoko.5v@kyoto-u.ac.jp (N.Y.); temple@kais.kyoto-u.ac.jp (M.T.); okumoto3@kais.kyoto-u.ac.jp (Y.O.); morisaka@kais.kyoto-u.ac.jp (H.M.); 2 Center for Medical, Agricultural, and Veterinary Entomology, Agricultural Research Service, USDA, 1600 S.W. 23RD Drive, Gainesville, FL 32606, USA; E-Mails: alisa.huffaker@ars.usda.gov (A.H.); Eric.Schmelz@ars.usda.gov (E.A.S.); 3 Department of Agriculture, Tottori University, Koyama-machi 4-101, Tottori 680-8550, Japan; E-Mail: aishihara@muses.tottori-u.ac.jp

**Keywords:** plant-insect interaction, plant induced resistance, insect-produced elicitors, isoflavones, insect herbivory, secondary metabolites, liquid chromatography, mass spectrometry

## Abstract

In response to attack by bacterial pathogens, soybean (*Gylcine max*) leaves accumulate isoflavone aglucones, isoflavone glucosides, and glyceollins. In contrast to pathogens, the dynamics of related insect-inducible metabolites in soybean leaves remain poorly understood. In this study, we analyzed the biochemical responses of soybean leaves to *Spodoptera litura* (Lepidoptera: Noctuidae) herbivory and also *S*. *litura* gut contents, which contain oral secretion elicitors. Following *S*. *litura* herbivory, soybean leaves displayed an induced accumulation of the flavone and isoflavone aglycones 4’,7-dihyroxyflavone, daidzein, and formononetin, and also the isoflavone glucoside daidzin. Interestingly, foliar application of *S*. *litura* oral secretions also elicited the accumulation of isoflavone aglycones (daidzein and formononetin), isoflavone 7-*O*-glucosides (daidzin, ononin), and isoflavone 7-*O*-(6’-*O*-malonyl-β-glucosides) (malonyldaidzin, malonylononin). Consistent with the up-regulation of the isoflavonoid biosynthetic pathway, folair phenylalanine levels also increased following oral secretion treatment. To establish that these metabolitic changes were the result of *de novo* biosynthesis, we demonstrated that labeled (^13^C_9_) phenylalanine was incorporated into the isoflavone aglucones. These results are consistent with the presence of soybean defense elicitors in *S*. *litura* oral secretions. We demonstrate that isoflavone aglycones and isoflavone conjugates are induced in soybean leaves, not only by pathogens as previously demonstrated, but also by foliar insect herbivory.

## 1. Introduction

Isoflavones play an important role in the defense response of soybean (*Glycine max* (L.) Merr.) to pathogen attack. When challenged with either bacteria (*Pseudomonas syringae* pv *glycinera*, *Ps*. *pisi*) or fungi (*Phytophthora megasperma* f. sp. *glycinea* (Hildeb), *Sclerotinia sclerotiorum* (Lib.) de Bary), soybean leaves accumulate isoflavones: daidzein, formononetin, isoformononetin, genistein, glycitein, and glyceollins I–III [1,2,3]. The induced isoflavonoids, particularly glyceollins, have been proposed as factors responsible for soybean resistance to several microorganisms [4,5]. In addition to these aglycones, three isoflavone glucosides (daidzin, genistin, ononin) and the malonylglucoside conjugates (malonyldaidzin and malonylgenistein) are also induced in soybean leaves following pathogen inoculation [3,6,7,8,9]. Soybean cultivars differ in concentrations of isoflavones and in their ability to accumulate isoflavones after the infection [3].

While microbial pathogens are more commonly examined, plants have sophisticated defense systems to perceive a broad range of biotic threats. This includes defense against herbivorous insects, which is mediated in part by the production of toxic and repellent compounds that accumulate after insect attack [10,11]. For example, maize and wheat respond to herbivory by accumulating the highly reactive benzoxazinoid, 2-β-D-glucopyranosyloxy-4,7-dimethoxy-1,4-benzoxazin-3-one glucoside (HDMBOA-Glc), which, upon liberation of free HDMBOA by plant β-glucosidases, is recalcitrant to herbivore detoxification and suppresses insect growth [12,13,14,15,16]. In a similar fashion, insect damage in cruciferous plants results in the myrosinase mediated hydrolysis of glucosinolates to produce highly toxic isothiocyanates that deter non-adapted herbivores [17,18]. Upon damage to developing seeds caused by southern green stink bug (*Nezara viridula*) feeding, multiple soybean genotypes locally accumulate elevated levels of flavonoids including daidzin and genistin [19]. Outside of this investigation in seed pods, the dynamics of insect-inducible metabolites in soybean leaves remain poorly understood.

In this study, we analyzed the biochemical responses of soybean leaves to *Spodoptera litura* (Lepidoptera: Noctuidae) herbivory. During attack by Lepidoptera larvae, trace levels of specific elicitors present in the insects oral secretions contact the wounded leaf surface and play an important role in amplifying induced plant defense responses [20,21,22,23,24]. To partly mimic and control this response, we also analyzed metabolites induced in soybean leaves treated with *S*. *litura* gut content which is known contain a mixture of oral secretion elicitors.

## 2. Results

### 2.1. Metabolites Induced by *S. litura* Herbivory

**Figure 1:**
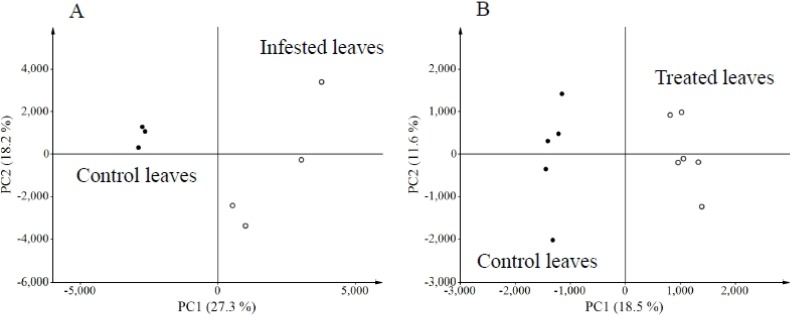
Principle Component Analyses (PCA) reveals the presence of herbivore and herbivore elicitor induced metabolites in soybean leaves. (**A**) PCA score plots of control and *S. litura*-infested leaves and (**B**) control and *S. litura* gut content treated leaves.

**Figure 2:**
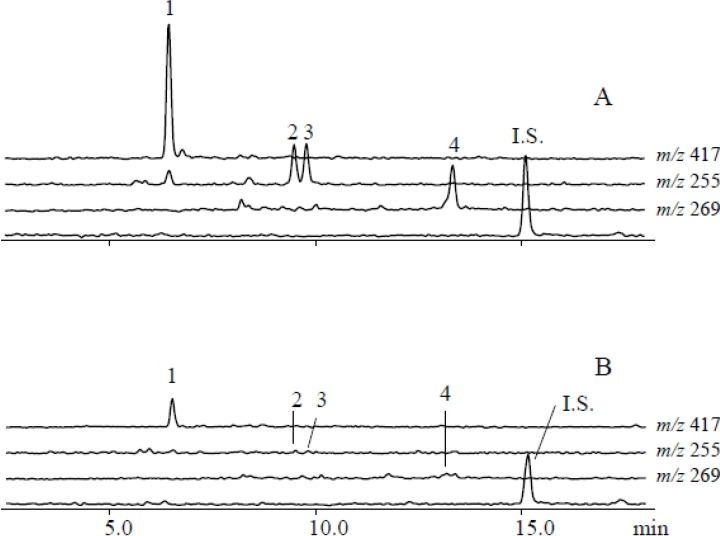
Qualitative analysis of soybean leaf flavonoids that accumulate following *S. litura* herbivory. LCMS [M+H]^+^ selected ion chromatogram profiles of (**A**) *S. litura* challenged and (**B**) control leaf extracts. Peak numbers (1–4) correspond to analytes as follows: (1) daidzin ([M+H]^+^, *m/z* 417); (2) 4’,7-dihydroxyflavone ([M+H]^+^, *m/z* 255); (3) daidzein ([M+H]^+^, *m/z* 255); and (4) formononetin ([M+H]^+^, *m/z* 269).

**Figure 3:**
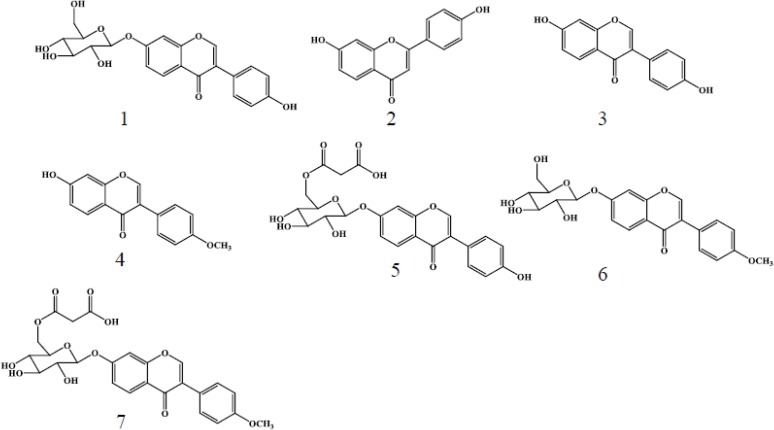
*S. litura* herbivory and gut content elicitation of soybean leaves results in an array of induced flavonoids. Structures and reference numbers (1–7) of compounds identified in the current study are as follows: (1) daidzin; (2) 4’,7-dihydroxyflavone; (3) daidzein; (4) formononetin; (5) malonyldaidzin; (6) ononin; and (7) malonylononin.

Using a metabolomics approach, we analyzed uninfested (control) and *S*. *litura*-infested plants using LCMS to identify herbivore-induced metabolites in soybean leaves. The numbers of metabolites projecting to principal component (PC) analyses were 15,990 for the herbivory treatment and 10,703 for the gut contents treatment. Consistent with a dynamic plant response, PC analyses revealed that control and infested leaves were clearly separated along the first PC axis (Figure 1A). PC loadings revealed that among all detected ions, a small subset contributed significantly to the metabolic difference between control and infested plants (Supplemental Figure 1 and Supplemental Table 1). To further evaluate candidate analytes that displayed differences between control and herbivory treatments, we analyzed soybean leaf extracts by LCMS 2020. We detected, five predominant proton adduct ions ([M+H]^+^ (*t*_R_ min): 1, *m*/*z* 417 (6.5 min); 2, *m*/*z* 255 (9.5 min); 3, *m*/*z* 255 (9.8 min); 4, *m*/*z* 269 (13.4 min) associated with infested leaves (Figure 2A) that were largely absent from control leaves (Figure 2B). These induced compounds were identified as daidzin (1), 4’,7-dihydroxyflavone (2), daidzein (3), and formononetin (4), respectively (Figure 3) by comparison of retention times and exact mass with authentic samples (Supplemental Table 2). Regarding 4’,7-dihydroxyflavone (2), daidzein (3), and formononetin (4) fragmentation patterns were further investigated by LC/Qtrap-MS and compared with authentic samples (Supplemental Figure 2A–C and Supplemental Table 3).

**Figure 4:**
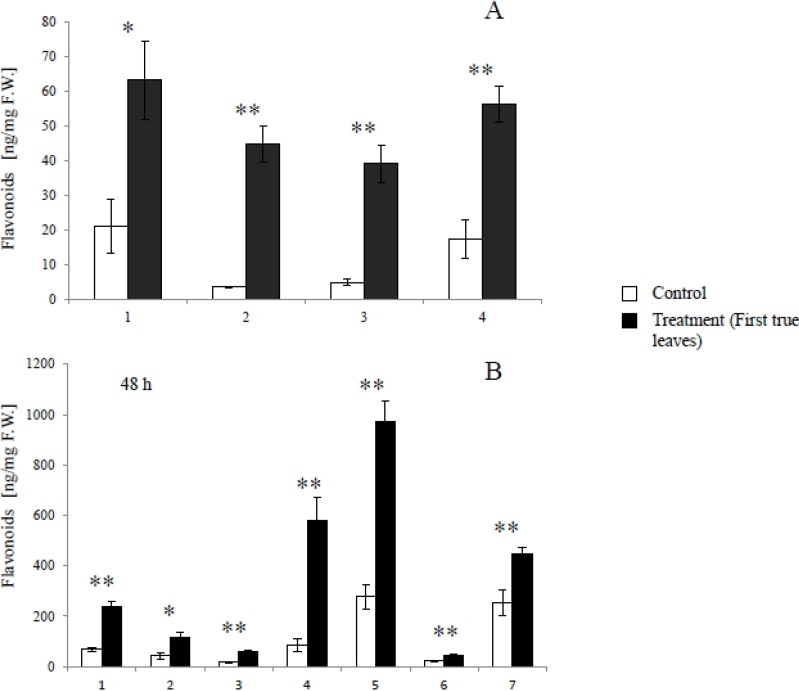
Quantitative estimates of soybean leaf flavonoids induced by herbivore and herbivore gut content elicitation. Average (n = 3–4; ± S.E.M.) flavonoid levels in soybean leaves challenged with (**A**) *S. litura* herbivory and (**B**) *S. litura* gut contents. (1) Daidzin; (2) 4’,7-Dihydroxyflavone; (3) Daidzein; (4) Formononetin; (5) Malonyldaidzin; (6) Ononin; (7) Malonylononin. The data were analyzed using the Student’s *t*-test and asterisks denote significant differences (*, *p* < 0.05; **, *p* < 0.01) relative to controls.

**Figure 5:**
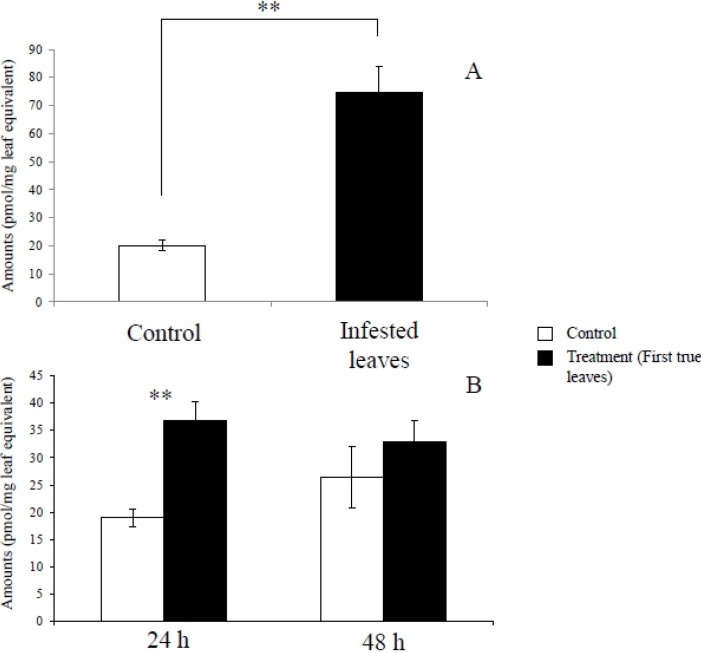
Amounts of induced phenylalanine in soybean leaves infested by *S. litura* larvae in 24 hours after the removal of larvae (**A**) and treated with gut contents of *S. litura* (**B**) in 24 and 48 hours after the treatment. The data were analyzed using the Student’s *t*-test and are presented as the mean ± S.E.M. (n = 3–4). Asterisks indicate significant differences (*, *p* < 0.05; **, *p* < 0.01) relative to the control.

Quantitative analysis showed that daidzin (1), 4’,7-dihydroxyflavone (2), daidzein (3), and formononetin (4) significantly accumulated in *S*. *litura*-infested leaves (first true leaves), compared to leaves of uninfested control plants (Figure 4A) 24 hours after the removal of the larvae. As a precursor of flavonoids, phenylalanine was also induced in the infested leaves (Figure 5A).

### 2.2. Metabolites Induced by *S. litura* Gut Contents

**Figure 6:**
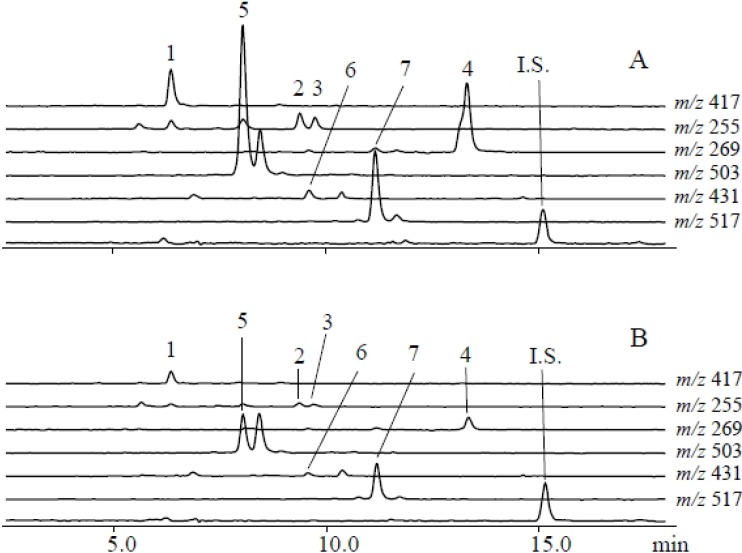
Elicitation of soybean leaves with *S. litura* gut contents results in a more complex induced flavonoid profile. Representative LCMS [M+H]^+^ selected ion chromatograms of soybean leaves 48 hours after treatment with either (**A**) *S. litura* gut contents or (**B**) buffer only controls. Flavonoid analytes are as follows: (1) daidzin ([M+H]^+^, *m/z* 417); (2) 4’,7-dihydroxyflavone ([M+H]^+^, *m/z* 255); (3) daidzein ([M+H]^+^, *m/z* 255); (4) formononetin ([M+H]^+^, *m/z* 269); (5) malonyldaidzin ([M+H]^+^, *m/z* 503); (6) ononin ([M+H]^+^, *m/z* 431); and (7) malonylononin ([M+H]^+^, *m/z* 517).

Gut contents of *S. litura* were solubilized in phosphate buffer and applied to mechanically damaged soybean leaves. Control leaves were similarly damaged and treated with phosphate buffer alone. LCMS analyses of gut content treated leaves followed from the *S. litura* herbivory experiments and were similarly analyzed by PC analyses (Supplemental Table 1). Gut content treated leaves and controls were clearly separated along first PC axis (Figure 1B). More detailed analyses of LCMS data were performed by loading plot (Supplemental Figure 3). Seven proton adduct ions ([M+ H]^+^ (*t*_R_ min): 1, *m*/*z* 417 (6.5 min); 2, *m*/*z* 255 (9.5 min); 3, *m*/*z* 255 (9.8 min); 4, *m*/*z* 269 (13.4 min); 5, *m/z* 503 (8.0 min); 6, *m/z* 431 (9.7 min); 7, *m/z* 517 (10.9 min) predominated in the analysis of treated leaves (Figure 6). The compounds corresponding to [M+H]^+^ ions 1–7 were identified as daidzin (1), 4’,7-dihydroflavone (2), daidzein (3), formononetin (4), malonyldaidzin (5), ononin (6) and malonylononin (7), respectively (Figure 3).

Malonyldaidzin (5) was identified by comparison of retention times (measured by nano-LCMS) and exact mass with an authentic sample. Ononin (6) and malonylononin (7) were identified by orbitrap-MS and NMR analyses.

As shown in Figure 4B, daidzin (1), 4’,7-dihydroxyflavone (2), daidzein (3), formononetin (4), malonyldaidzin (5), ononin (6), and malonylononin (7) accumulated in soybean leaves 48 hours after treatment gut contents. Phenylalanine significantly increased in 24 hours, although there was no significant difference in the amount of phenylalanine in 48 hours between the treatment and the control (Figure 5B).

### 2.3. Gut Content Extracts Treatment with Labeled Phenylalanine

**Figure 7:**
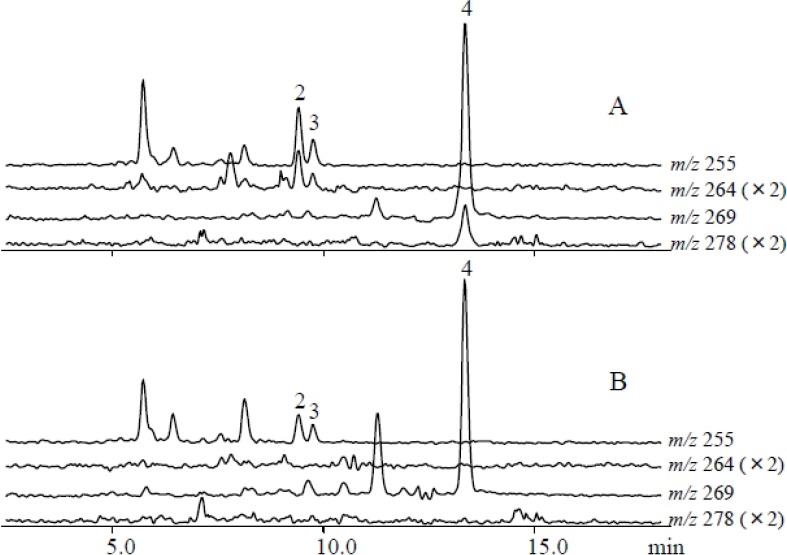
Evidence for local isotopic incorporation of ^13^C_9_-phenylalanine into soybean leaf flavonoids elicited by *S. litura* gut contents. LCMS ([M+H]^+^ selected ion chromatograms of ^13^C-labeled (2) 4’,7-dihydroxyflavone, (3) daidzein, and (4) formononetin (4) from leaves treated with (**A**) gut content extracts plus ^13^C_9_-phenylalanine compared to (**B**) control leaves. ^13^C_9_-phenylalanine incorporation into flavonoids was detected as an increased isotopic abundance of 9 Da into 4’,7-dihydroxyflavone ([M+H]^+^, *m/z* 264) and daidzein ([M+H]^+^, *m/z* 264), and formononetin ([M+H]^+^, *m/z* 278). Non-labeled 4’,7-dihydroxyflavone, daidzein, and formononetin are detected at *m/z* 255, *m/z* 255, and *m/z* 269, respectively, as shown in Figure 2.

Soybean leaves 48 hours after the treatment with gut content extracts containing ^13^C_9_-phenylalanine (5 μg/μL) were analyzed by LCMS. [M+H]^+^ ions of *m/z* 264 (corresponding to labeled ions of 4’,7-dihydroxyflavone and daidzein) and 278 (corresponding to labeled ions of formononetin) were detected at the same retention times of each non-labeled ions (Figure 7). Ions of labeled flavonoids aglycones were not detected from control leaves. This supports our quantitative data that leaf treatment with gut content extracts activates the biosynthetic pathway from phenylalanine to flavonoid aglycones.

## 3. Discussion

We identified daidzin (1), 4’,7-dihyroflavone (2), daidzein (3), and formononetin (4) as *S*. *litura* inducible metabolites following herbivory in soybean leaves. Additionally malonyldaidzin (5), ononin (6), and malonylononin (7) were also accumulated in soybean leaves treated with *S*. *litura* gut content extracts. Given that many analytes were present at 3–10 fold greater levels in leaves treated with gut contents compared to *S*. *litura* herbivory, we suspect that the additional detection of malonyldaidzin (5), ononin (6), and malonylononin (7) occurred due to a greater overall level of rapid elicitation. These conjugates are considered to be latent forms of isoflavonoids and upon cleavage are ultimately converted to the aglycones, daidzein (3) and formononetin (4) [25]. Another possibile difference between *S*. *litura* herbivory and application of gut contents is that soybean isoflavone conjugate-hydrolyzing β-glucosidases may exhibit greater activation in soybean leaves damaged by continuous herbivory compared to a single damage time point. In general the induced defense responses of soybean leaves to *S*. *litura* herbivory were largely reproduced by application of *S*. *litura* gut content extracts.

When damaged by *S*. *exigua*, corn plants release volatile compounds which can serve as host location cues for natural enemies of the herbivores. Synthesis and release of plant volatiles is elicited by specific substances present in herbivore oral secretions and gut contents. The best known of these insect-produced elicitors are the fatty acid amino acid conjugates (FACs), first identified from *S*. *exgua* larvae [20]. The FACs are also found in several other lepidopteran species [26,27,28,29,30,31,32,33]. FACs also induce soybean to release volatile compounds [34] and tobacco plants to produce trypsin proteinase inhibitors [23]. We add to this body of knowledge by demonstrating that elicitors present in *S*. *litura* gut contents induce the production of flavonoid biosynthesis in soybean leaves. The future identification of *S*. *litura* elicitors responsible for this response will further contribute to our understanding of soybean defense regulation.

Furthermore, it is important to investigate whether these induced compounds are accumulated locally or systemically in response to the insect feeding and elicitor treatment. For example, cotton plants *Gossypium hirsutum* L. damaged by herbivorous insects release volatiles, which are released, not only at the damaged site, but from the entire cotton plant [35]. All systemically released compounds are known to be induced by caterpillar feeding. Larval oral secretions of *Manduca sexta* or synthetic FACs significantly increase trypsin proteinase inhibitor elicitation in both treated (local) and systemic untreated leaves in *Nicotiana attenuata* [23]. Further studies are necessary to evaluate whether these induced flavone and isoflavones are produced locally or systemically following the treatments in the near future.

Nicotine, an inducible defense compound in *Nicotiana* species, is produced in the roots after insect herbivory and mechanical damage, and then transported to aerial parts of the plant [36]. In soybean roots, significant levels of daidzin (1), daidzein (3), and malonyldaizin (5), are also reported [7]. To investigate whether or not flavonoid synthesis occurs at the site of elicitation, we applied ^13^C-labeled phenylalanine to damaged leaves in the presence of gut content elicitors and searched for isotopic incorporated into daidzein (3) and formononetin (4). As shown in Figure 7, labeled phenylalanine was incorporated into 4’,7-dihydroxyflavone (2), daizdzein (3), and formononetin (4) 48 hours after the elicitor treatment.

When subjected to bacterial or fungal challenge, soybean leaves also accumulate glyceollins [1,4,5,37,38,39,40]. No increase in the aglycones, the glucoside conjugates, the malonylglucoside conjugates, or glyceollins occurs after leaf tissues are injured by physical stress [2]. Given that the accumulation of glyceollins are believed to play a significant role in the resistance of soybean cultivars to *Phytophthora megasperma* [4,5] and that daidzein (3) is a biosynthetic precursor of glyceollins [38], regulation of the daidzein is important early step in disease resistance. Similar to established pathogen induced responses, *S*. *litura* attack on soybean leaves results in the accumulation of daidzein (3) and formononetin (4). In contrast to pathogen elicitation, glyceollins have not been identified in leaves damaged by *S*. *litura* or those treated with gut contents, while glyceollins are reported to accumulate in soybean (var. Tamahomare) cotyledons [41] treated with one of polysaccharides, such as elicitors derived from fungal cell wall [42]. These results support the accumulation of daidzein (3) and formononetin (4), but not glyceollins, following *S*. *litura* herbivory. This selective plant response is likely to be important defensively as Zhou *et al.* demonstrated that daidzein can significantly inhibit the larval growth of *S*. *litura* [43]. Although no direct functions of the inhibitory activity of formononetin (4) against *S*. *litura* larvae has also been reported thus the accumulation of daidzein (3), without transference to glyceollins, likely represents an adaptive anti-herbivore defensive strategy of soybean.

In nature, plants are constantly challenged by a multitude of biotic stresses, including herbivorous insects and phytopathogens. However, most studies focus on responses to a single attacker and without seeking to understand synergies and trade-offs between insect and pathogen resistance in plants. In the present study we demonstrate that, in additional to established responses to pathogens, soybean leaves also synthesize and accumulate a subset of flavonoids following herbivore attack. Future investigations will include the analysis of gene expression and biosynthetic enzymes to better understand the role of flavonoid defenses against diverse numerous biotic attackers that operate in terrestrial ecosystems both above and belowground.

## 4. Materials and Methods

### 4.1. Plants and Insects

Soybean (cultivar Tamahomare) seeds were obtained from Nagano Prefecture Vegetable and Ornamental Crops Experiment Station and germinated in clay pots containing vermiculite, fertilized with 0.2 g/kg N, 0.4 g/kg P, and 0.3 g/kg K. All plants were grown in a greenhouse under natural light conditions with temperature 15–30 °C. Common cutworm *Spodoptera litura* larvae (Lepidoptera: Noctuidae) were reared on artificial diet (Insecta-LFS, Nihon Nosan Kogyo Ltd., Yokohama), at 24 °C under a 16 h/ 8 h (light/dark) cycle.

### 4.2. Chemicals

Daidzein and formononetin were purchased from LKT laboratories, Inc (St. Paul, MN, USA). An isoflavone aglycone mixture from soybean (including daidzin and malonyldaidzin), 4’,7-dihydroxyflavone, and 2’-hydroxyflavanone were purchased from Wako Pure Chemical Industries, Ltd (Osaka, Japan), Extrasynthese (Genay, France), and Tokyo Chemical Industry (Tokyo, Japan), respectively.

### 4.3. Feeding Treatment

Five *S*. *litura* larvae (3rd instar) were placed on the first true leaves of soybean plant in V3 stage, where the second true leaves were fully developed. The leaves were covered in a polyethylene (PET) bag to stop the larvae from escaping or left herbivore-free (n = 3) overnight. The damaged first true leaves and the undamaged control true leaves were extracted and analyzed by LCMS (detailed below) 24 hours after the removal of the larvae.

### 4.4. Gut Contents Treatment

Thirty final instar *S*. *litura* larvae were anesthetized by immersion in tepid water to 10 min and then dissected in saline. Gut (crop and midgut) contents were obtained by slitting the gut tube which was removed from the larvae. Gut contents were homogenized in 50 mM Na_2_HPO_4_-NaH_2_PO_4_ buffer (6 mL, pH 8) and then heated at 80 °C for 20 min. After centrifugation at 12,000 *g* for 5 min, the supernatant extracts were used for gut content treatments. Using V3 stage soybean plants, approximately 25 pin holes were created in area of the center of the central trifoliate of the first true leaves and then 10 μL of gut content extracts (0.05 larval equivalents) were applied on the holes. Leaves were harvested 24 and 48 hours later, extracted and analyzed by LC/MS, as shown below.

### 4.5. Phenylalanine Derivatization

Phenylalanine was derivatized with 6-aminoquinolyl-*N*-hydroxy-succinimidyl carbamate (AQC) as described previously with modification [44]. Ten microliters of leaf extracts were mixed with 60 μL of 200 mM borate buffer (pH 8.8) and 10 μL of 50 μM of β-alanine in water. An aliquot of AQC-acetonitrile (20 μL of 3 mg/mL) was then added and immediately vortexed for 1 min. The samples were heated to 55 °C for 10 min, centrifuged at 12,000 *g* for 5 min, and filtered supernatants were then analyzed by LCMS (Shimadzu 2020, Kyoto, Japan).

### 4.6. Extraction and Sample Preparations

Soybean leaves were extracted with 80% MeOH solution (MeOH/H_2_O, v/v) (0.2 mg fresh weight/μL), which contained 2’-hydroxyflavanone (50 ng/μL) as internal standard, and pulverized with a bead homogenizer. After centrifugation at 3000 *g* for 5 min, the supernatants were filtered and samples (1 μL) were analyzed by LCMS systems (2020, Shimadzu and 4000 Qtrap, AB Sciex) and a 100 fold dilution (in H_2_O) of the extracts were analyzed by nanoLCMS (LTQ OrbitrapVelos, Thermo Fisher Scientific).

### 4.7. Instruments for Chemical Analysis

LCMS was carried out using an LCMS-2020 equipped with Prominence HPLC system (Shimadzu, Kyoto, Japan) in ESI positive ion mode. A reversed- phase column (Mightysil RP-18 GP2.0 X 50 mm I.D., Kanto Chemical Co., Inc, Tokyo, Japan) was eluted (0.2 mL/min) with gradient of 5%–50% (0–13 min), 50%–99% (13–20 min) acetonitrile containing 0.08% acetic acid in water containing 0.05% acetic acid. For amino acid analysis, the same column was eluted with gradient of 1% (0–2 min), 1%–15% (2–9 min), 15%–30% (9–14 min), 30%–80% (14–20 min) acetonitrile containing 0.1% formic acid in water containing 0.1% formic acid. The column temperature was maintained at 40 °C. The MS was operated with nebulizer gas flow of 1.5 L/min, drying gas flow of 15 L/min, ESI voltage of 1.8 kV, temperature of 250 °C.

LCMSMS analysis was performed by a mass spectrometry (4000 Qtrap, AB Sciex) combined with the Shimadzu HPLC system, as shown above. The column and gradient patterns are the same as previously mentioned. The MS was operated with ESI voltage of 5.5 kV, nebulizer gas flow of 50 L/min, turbo gas flow of 80 L/min, gas temperature of 350 °C, CE of 40 eV.

Exact mass analysis was performed by a nano-liquid chromatography (Ultimate 3000, DIONEX)-mass spectrometry system (LTQ OrbitrapVelos, Thermo Fisher Scientific). Extracts (2 μL) were injected and separated by reversed-phase chromatography using a monolithic column (1000 mm, 0.1 mm I.D., Kyoto Monotech Co., Ltd., Kyoto, Japan) at flow rate of 1.0 μL/min. The gradient was 10% (0–10 min), 10%–99% (10–40 min), 99%–100% (40–45 min) 80% acetonitrile/water containing 0.1% formic acid in water containing 0.1% formic acid. The MS was operated with ESI voltage of 2.3 kV, the transfer tube temperature of 280 °C. Reference standard of daidzin, 4’,7-dihydroxyflavone, daidzein, formononetin, and malonyldaidzin were detected at 24.9, 29.8, 30.2, 35.2, and 27.1 min. [M+H]^+^ Ions of daidzin, 4’,7-dihydroxyflavone, daidzein, formononetin malonyldaidzin, ononin, and malonylononin were observed at *m*/*z* 417.121 (M^+^ 416.113; calculated 416.111; C_21_H_20_O_9_), 255.068 (M^+^ 254.060; calculated 254.058; C_15_H_10_O_4_), 255.068 (M^+^ 254.060; calculated 254.058; C_15_H_10_O_4_), 269.083 (M^+^ 268.075; calculated 268.074; C_16_H_12_O_4_), 503.122 (M^+^ 502.114; calculated 502.111; C_24_H_22_O_12_), 431.138 (M^+^ 430.130; calculated 430.126; C_22_H_22_O_9_), and 517.138 (M^+^ 516.131; calculated 516.127; C_25_H_24_O_12_).

### 4.8. Preparation of Ononin and Malonylononin

Fresh leaves of soybean were extracted with 80% aqueous methanol. The extract was evaporated to dryness and dissolved in 5% aqueous methanol. The solution was separated into 4 fractions on a Waters Sep-Pak Vac (10 g) C18 cartridge, using step gradients of methanol-water mixtures (fr. 1, 5% methanol; fr. 2, 15% methanol; fr. 3, 30% methanol; fr. 4, 50% methanol; 60 mL each). Fraction 4 was re-chromatographed on a reverse phase HPLC column (Mightysil RP-18GP, 250 × 10 mm I.D., Kanto Chemical Co., Inc, Tokyo, Japan) with gradient of 5%–23% (0–10 min), 23%–30% (10–30 min), 32%–99% (30–35 min) acetonitrile containing 0.08% acetic acid in water containing 0.05% acetic acid at a flow rate of 2.0 mL/min to purify ononin and malonylononin.

Proton nuclear magnetic resonance (^1^H-NMR) spectra were measured with Bruker AV-400 III Spectrometer (400 MHz) using TMS as an internal standard. Ononin (formononetin-7-*O*-glucoside): ^1^H-NMR (400 MHz, CD_3_OD) δ_H_: 8.25 (1H, s, H-2), 8.16 (1H, d, *J* = 8.8 Hz, H-5), 7.50 (2H, d, *J* = 8.8 Hz, H-2’, 6’), 7.27 (1H, d, *J* = 2.3 Hz, H-8), 7.22 (1H, dd, *J* = 2.3, 8.8 Hz, H-6), 7.01 (2H, d, *J* = 8.8 Hz, H-3’, 5’), 5.11 (1H, d, *J* = 7.6 Hz, H-1’), 3.94 (1H, dd, *J* = 2.2, 11.9 Hz, H-6’a), 3.86 (3H, s, OCH_3_), 3.75 (1H, dd, *J* = 6.0, 11.9 Hz, H-6’b), 3.6 to 3.4 (4H, m, H-2’-5’). Malonylononin (formononetin-7-*O*-(6’-*O*-malonylglucoside)): ^1^H-NMR (400 MHz, CD_3_OD) δ_H_: 8.26 (1H, s, H-2), 8.17 (1H, d, *J* = 8.9 Hz, H-5), 7.51 (2H, d, *J* = 8.8 Hz, H-2’, 6’), 7.28 (1H, d, *J* = 2.2 Hz, H-8), 7.22 (1H, dd, *J* = 2.2, 8.9 Hz, H-6), 7.01 (2H, d, *J* = 8.8 Hz, H-3’, 5’), 5.11 (1H, d, *J* = 7.6 Hz, H-1’ ), 4.57 (1H, dd, *J* = 2.1, 11.9 Hz, H-6’a), 4.28 (1H, dd, *J* = 6.8, 11.9 Hz, H-6’b), 3.86 (3H, s, OCH_3_), 3.6 to 3.4 (4H, m, H-2’-5’), 3.36 (2H, s, malonyl-H).

### 4.9. Statistical Analysis

LCMS data (*m/z* and retention times) was exported by using profiling solution^®^ software (Shimadzu). LCMS fingerprints of samples were processed using Profiling Solution 1.1 Build 104 (Shimadzu, Kyoto, Japan) for mass signal extraction and alignment from 5 to 15 min with *m/z* values from 200 to 600 Da with the following parameters: an ion m/z tolerance of 25 mDa, an ion retention time tolerance of 1.5 min, ion intensity threshold at 1000 counts, detecting 20% isomer valley and allowing some ions without isotope peaks. PCA was performed using SIMCA P (version 13.0.0.0), which is a multivariate data analysis software that uses soft independent modeling of class analogies (SIMCA). The data were analyzed using the Student’s *t*-test and are presented as the mean ± S.E.M. (n = 3–4). Asterisks indicate significant differences (*, *p* < 0.05; **, *p* < 0.05) relative to the control.

## Supplementary Files

Supplementary File 1
