# Supplementary material for: Insect-Induced Daidzein, Formononetin and Their Conjugates in Soybean Leaves
Source: Metabolites. 2014 Jul 4;4(3):532–46. doi: 10.3390/metabo4030532 (PMC4192678; doi:10.3390/metabo4030532)
Supplement: Supplementary File 1 [file metabolites-04-00532-s001.pptx]

## Slide 1
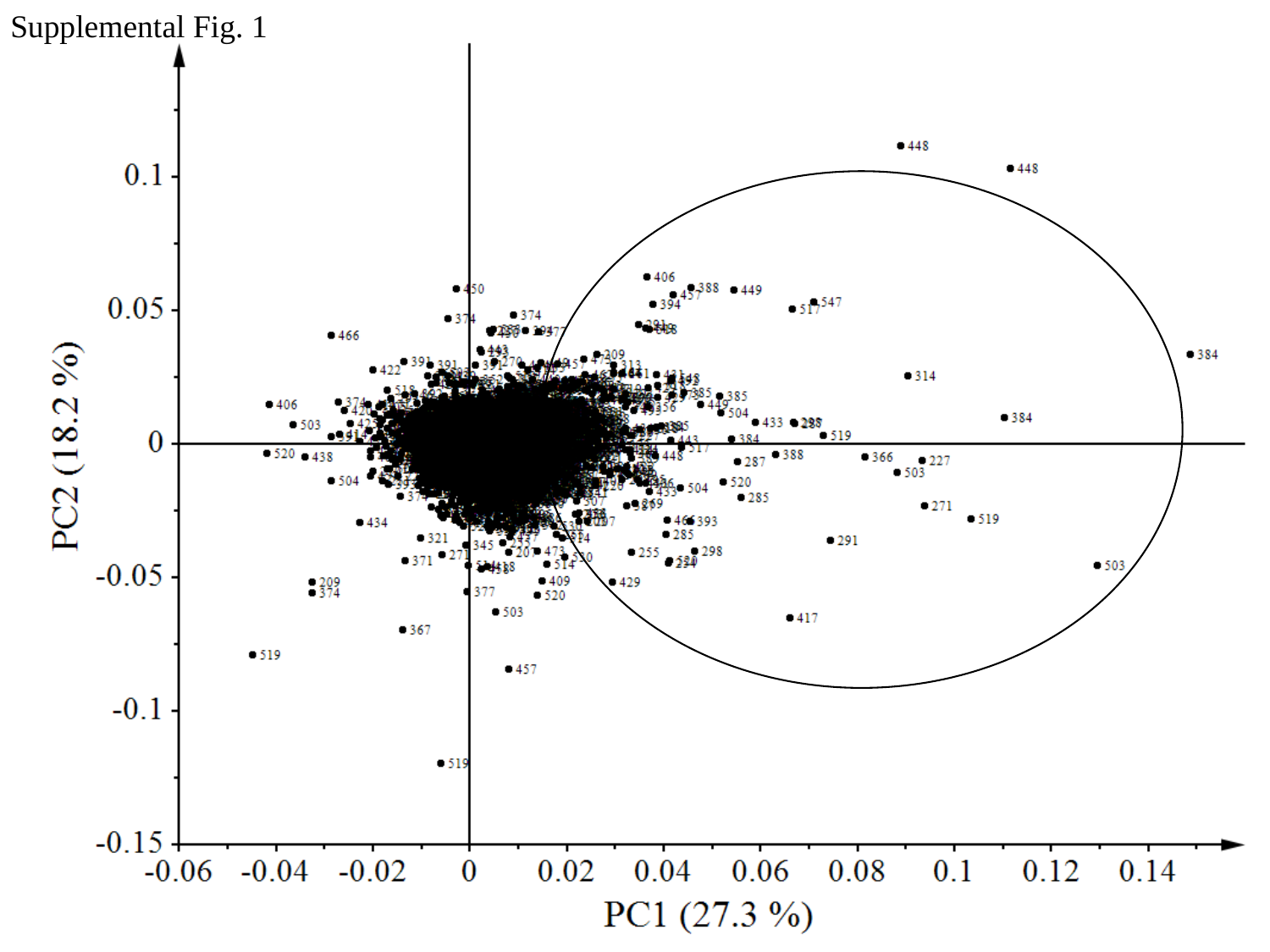

Supplemental Fig. 1

## Slide 2
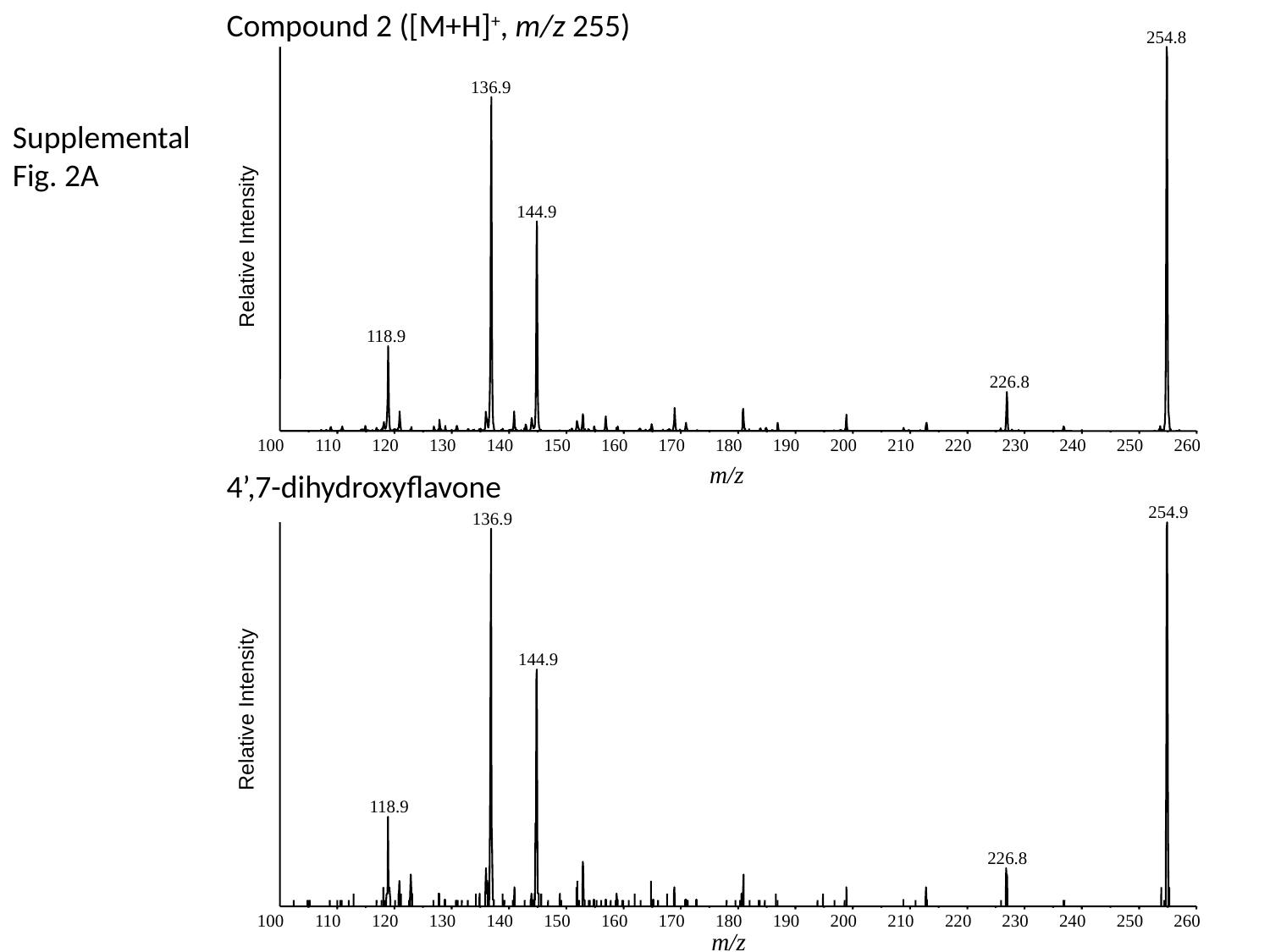

Compound 2 ([M+H]+, m/z 255)
254.8
136.9
Supplemental
Fig. 2A
Relative Intensity
144.9
118.9
226.8
100
110
120
130
140
150
160
170
180
190
200
210
220
230
240
250
260
m/z
4’,7-dihydroxyflavone
254.9
136.9
Relative Intensity
144.9
118.9
226.8
100
110
120
130
140
150
160
170
180
190
200
210
220
230
240
250
260
m/z

## Slide 3
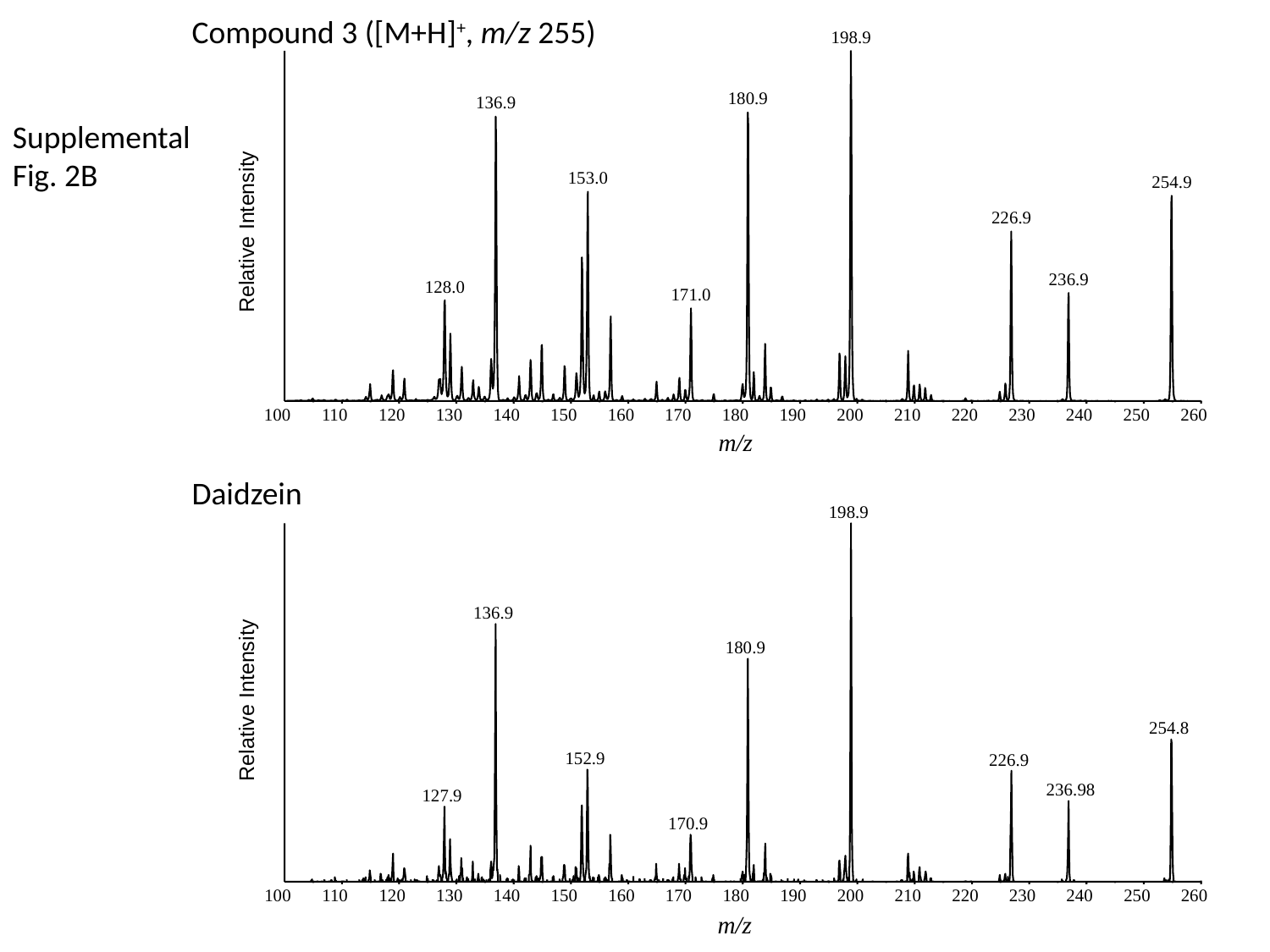

Compound 3 ([M+H]+, m/z 255)
198.9
180.9
136.9
Supplemental
Fig. 2B
Relative Intensity
153.0
254.9
226.9
236.9
128.0
171.0
100
110
120
130
140
150
160
170
180
190
200
210
220
230
240
250
260
m/z
Daidzein
198.9
136.9
Relative Intensity
180.9
254.8
152.9
226.9
236.98
127.9
170.9
100
110
120
130
140
150
160
170
180
190
200
210
220
230
240
250
260
m/z

## Slide 4
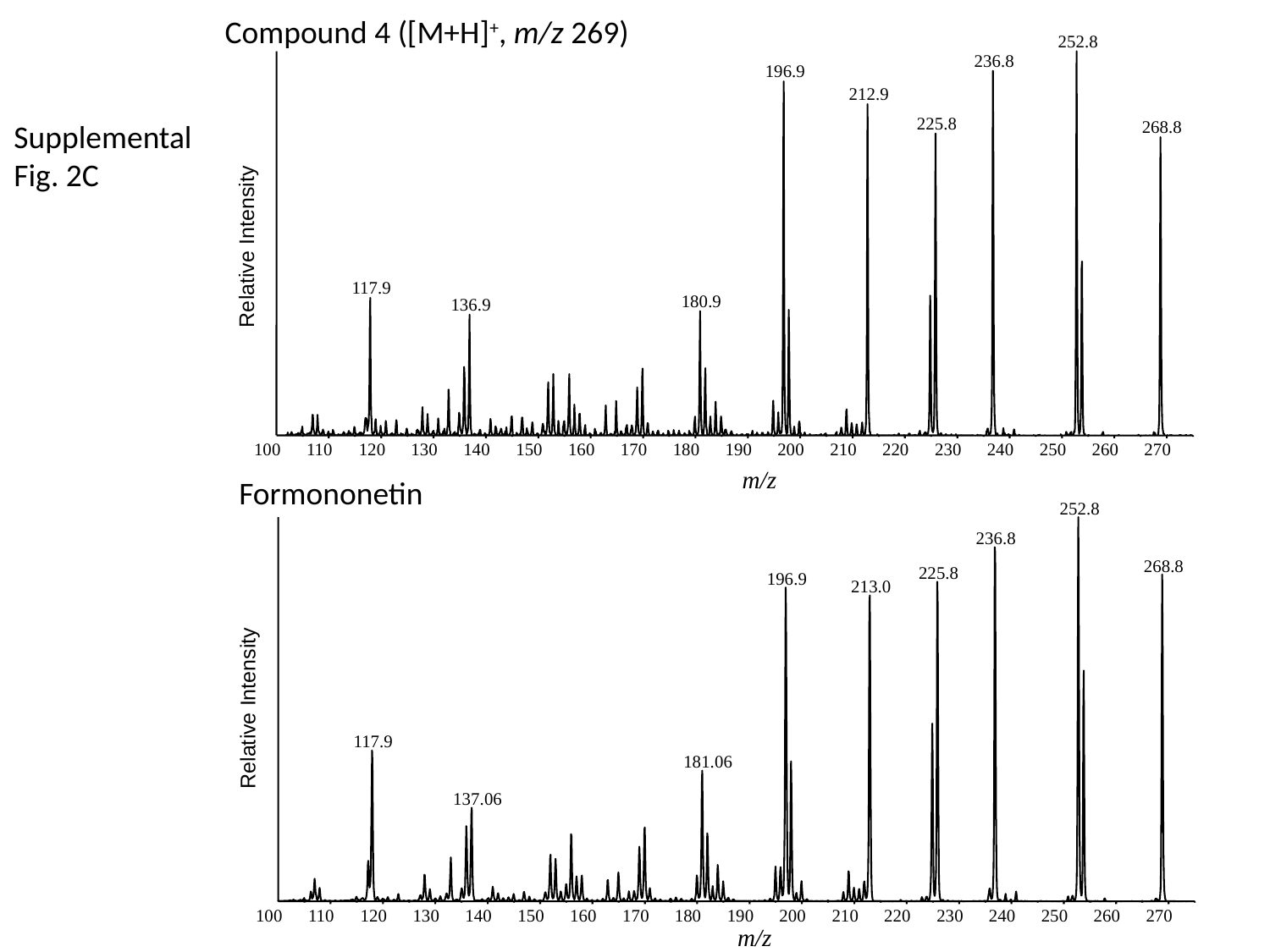

Compound 4 ([M+H]+, m/z 269)
252.8
236.8
196.9
212.9
Supplemental
Fig. 2C
225.8
268.8
Relative Intensity
117.9
180.9
136.9
100
110
120
130
140
150
160
170
180
190
200
210
220
230
240
250
260
270
m/z
Formononetin
252.8
236.8
268.8
225.8
196.9
213.0
Relative Intensity
117.9
181.06
137.06
100
110
120
130
140
150
160
170
180
190
200
210
220
230
240
250
260
270
m/z

## Slide 5
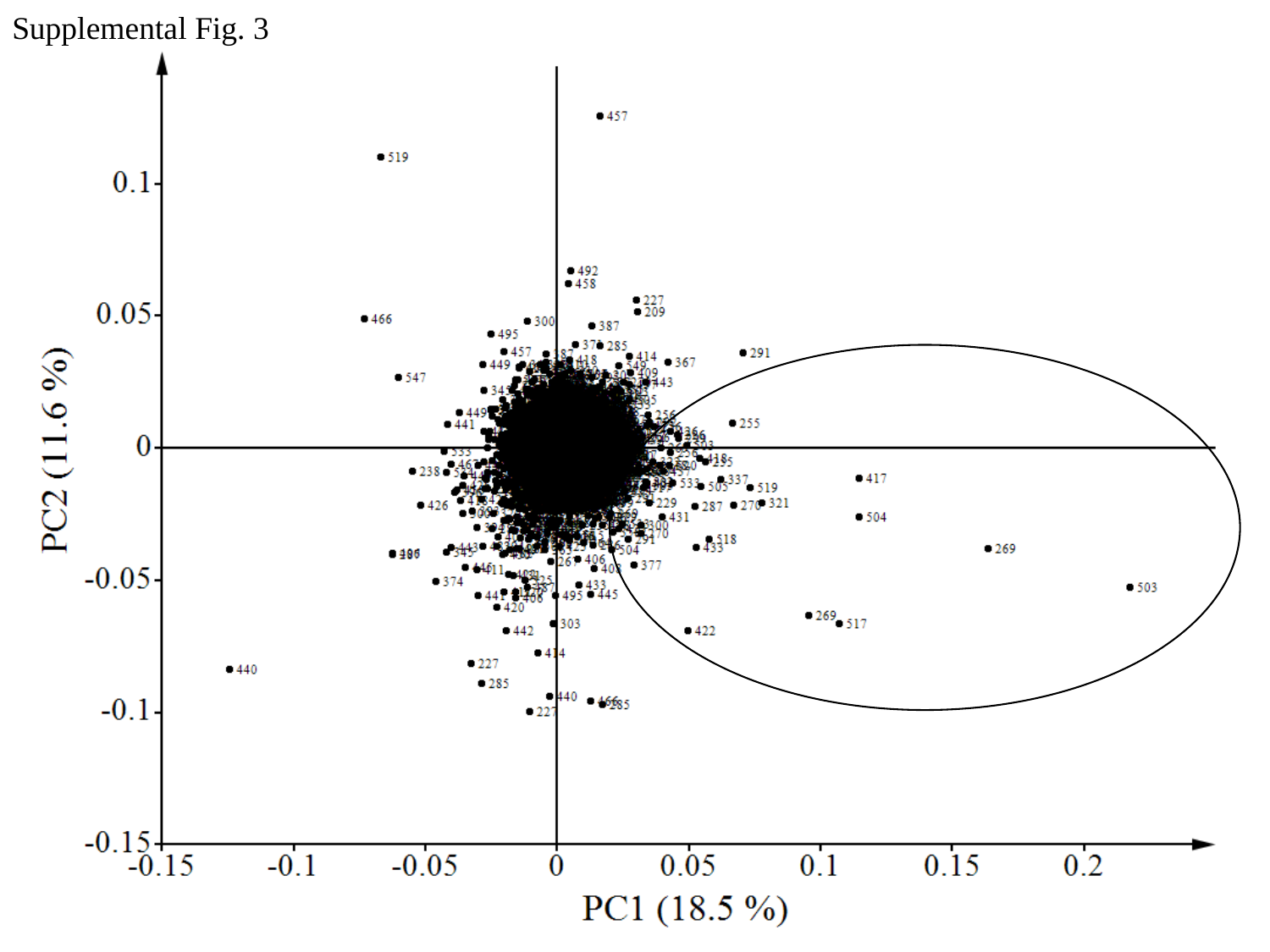

Supplemental Fig. 3
